# EDUCATIONAL INITIATIVE ABOUT MEDICATION MANAGEMENT FOR OLDER ADULTS

**DOI:** 10.1093/geroni/igz038.138

**Published:** 2019-11-08

**Authors:** Manpreet Boparai, Rosario Costas Muniz, Natalie Gangai, Ruth Manna, Beatriz Korc-Grodzicki

**Affiliations:** Memorial Sloan Kettering Cancer Center, New York, United States

## Abstract

Older adult patients often have multiple comorbidities, see multiple providers, leading to poor medication management (MM). Educational seminars were conducted to health care providers (HCPs) and community-based organizations to deliver health programs to linguistically diverse communities by a pharmacist utilizing interpreters. Participant knowledge was assessed using matched pre-post surveys (translated to participants’ respective languages). Behavioral intention was measured using a Likert scale. Results indicate participation from 136 community dwelling adults via 6 educational sessions; 44 caregivers via 5 sessions, and 22 HCPs (4 students and 22 residents) participated in one session. 91% of the community population were born outside of the US, 67% coming from South Asian countries, speaking over 7 different languages. Knowledge about medication management increased from an average of 44% correct responses pre-session to 48% correct post-session – a statistically significant change (t (135) =-2.26, p<.03). Most caregivers were females (82%) and born in a South Asian country (87%). Knowledge about medication management when caring for a loved one increased significantly from an average from pre (46%) to post (60%) (t (41) =-3.07, p< .01) Finally, most HCPs were females (53%), 63% Asian; 87% were born outside of the US, predominantly in East Asia. Providers had a significant increase on knowledge (t (21) =-3.03, p< .01) about prescribing for older adults. Detailed results of the programs will be presented. Despite challenges in diverse audience members, all three target populations indicated a statistically significant change in knowledge indicating the value in targeted and tailored health education topics.

